# This is who to blame for the COVID-19 pandemic

**DOI:** 10.7189/jogh.11.03035

**Published:** 2021-02-11

**Authors:** Igor Rudan

**Affiliations:** Centre for Global Health, Usher Institute, University of Edinburgh, Edinburgh, Scotland, UK

In the first phase of the pandemic, the virus was expected to enter other countries from China. The governments of those countries were getting prepared for that entry. Some took it more seriously, others less so. But at least the aim was pretty clear back then. Epidemiologists understood the threat and prepared their first line of defence. It consisted of identifying all those infected through testing and isolating all their contacts. This was going to prevent the virus from spreading freely among the population. The first line of defence was supposed to hold back the virus for as long as possible. In February 2020, most of the countries still hoped that this pandemic would be fully controllable in this way [1].

If that first line was breached, it was understood that the second phase would involve some kind of lockdown. There was simply no other form of defence strategy against the free spread of a new and unknown virus. Although experienced epidemiologists suspected that infection-fatality rate (IFR) may be in the range between 0.5%-1%, the only indication of severity at the time was case-fatality rate (CFR), which was much higher – it ranged between 3% and 6% in China, Iran and Italy. An evidence-based approach to medicine required that the decisions should be made upon CFR and wait until mid-April for seroprevalence tests before we could assess IFR. In March 2020, no-one had the right to gamble with the death toll based on their epidemiological intuition and CFR was all that was available [1,2].

Therefore, this second phase also had rather clear goals. The lockdown should have been implemented timely, at the first notice of free spread of the virus in the population. It should have been designed to last as short as possible, leaving few viruses in circulation after its completion. It would be used to learn more about the virus, illuminate its mode of transmission, learn about the speed of its spread, clarify the IFR instead of CFR through seroprevalence studies, understand age- and sex-specific risk of dying, study longer-term effects, educate the population accordingly, test the potential effectiveness of the existing drugs and approaches, accelerate vaccine development and identify better ways to prevent its spread than the lockdowns were. In addition, assistance to the companies in the private sector had to be provided to mitigate the impact on economic activities as much as possible [1-3].

After the lockdowns ended in most countries of the world that implemented them, the third phase of the pandemic began. It was clear that any new lockdowns would severely harm the economy. That is why this third phase was the most uncertain. There were no longer any clear goals. No-one wanted to go back to quarantine. But, on the other hand, no one wanted an uncontrolled epidemic, either [4].

The growing problem of this third phase is that people are not used to having their lives chronically stuck between two bad options. Individuals and groups, as well as entire societies, generally want to have a number of options, so that they can choose the best among them. Instead, we all found ourselves trapped between two bad options. Ignoring the virus causes casualties and threatens the function of health systems. Doing something to control it changes our lives for worse and threatens the economy. Little by little, people who are trapped between these two bad options will grow increasingly frustrated. Soon enough, they will start looking for the culprit [4].

But who is to blame for this situation? This is not an easy question to answer, because one must first understand that a billion people in the world are starving [5,6]. As a result, about nine million people worldwide die of hunger each year [7,8]. At the same time, the remaining 6 billion throw away 30% of the food they produce [8]. This means that there is surely enough food in the world today to feed the entire human population. However, about 9 million still die and further one billion starve because there is no global coordination to better distribute food among the world’s population [8].

As long as a billion hungry people continue to survive in huge countries that are still developing infrastructurally and economically, in any village of each of these nations a starving person could eat any local animal. This event alone could trigger another pandemic. Therefore, the prevention of future such pandemics won’t be ensured by simply closing wet markets in China or everyone’s switching to vegetarianism. The problem is much bigger and more complex. Namely, world hunger should be eradicated.

Another underlying cause that will favour future pandemics is that humans are intensely destroying the habitats of other species to expand their own space [9]. When populations of many other species that harbour viruses decline, while the human population is rapidly increasing, viruses will try to jump on us. They, too, aim to keep going, and it is becoming safer for them to use our species as their reservoir. Many species will die out because of our actions, but their viruses will try to find a new host and humans are very attractive. That is why new epidemics and pandemics await us in the future. We have already had several dangerous epidemics in the 21st century, but COVID-19 is the first to spread around the world and paralyze us to this extent [10].

The third reason why we found ourselves in this unpleasant situation is that humanity did not choose to invest significantly more in the prevention and be better prepared for the pandemic [10,11]. Many scientists could have worked for years on the development of coronavirus and other vaccines but investing in such projects is usually not profitable for any investors. The profit from such investments is extremely uncertain. Therefore, financial support to such projects should be made in good faith, with a vision of possible benefits in the future. The same is probably true for other unpredictable events — earthquakes, volcanic eruptions or even meteor strikes. We know that they would cause big problems in time and there are probably at least some research and development that we could get under way to mitigate their future damage. But few are willing to invest right now, given that we can’t tell when and where the defence might be needed.

However, if we consider how much money humanity has invested over the past half-century in the construction of its many settlements and roads, the production of cars and technical devices, or in the cosmetics industry, we could have certainly ensured sufficient funds to invest in preventing possible pandemics — especially from the new coronavirus, given that SARS and MERS had already caused a scare. But that hasn’t been done, at least not to a sufficient extent [10]. Unfortunately, as a result, our lives this year have been quite different from what we were used to.

The fourth underlying cause that exaggerates our current problems is the exposed fragility of national economies. We now learned that just a few weeks of retreating to homes and reducing personal consumption and spending can lead to economic declines that have not been recorded in recent history [12]. It also became quite clear how much economic inequality is there in today’s world [13]. Large parts of the population cannot survive even a few weeks of isolation and they have to continue to work to survive. These are just a few major insights on a global scale which this pandemic has revealed. Our civilization is still rather fragile, and it is sensitive to unforeseen stresses.

From these four global causes, we could move to the lessons learned by the individual countries. The first wave of the pandemic has taught us at least two important lessons. There were good examples of the nations that took the threat seriously and prepared very well for this crisis [2]. They were ready for the arrival of the virus and they resolutely prevented it from spreading. Some managed to even eradicate it from within their borders for extended periods of time. Those countries protected their citizens from the pandemic’s first wave and saved many lives. At the same time, they also generally managed to protect their economies from major shocks [2]. However, those countries that underestimated the danger of the epidemic recorded a very large number of deaths. Following such tragedies, they typically found themselves in prolonged lockdowns. In most of them, their economies suffered considerable blows [3].

**Figure:**
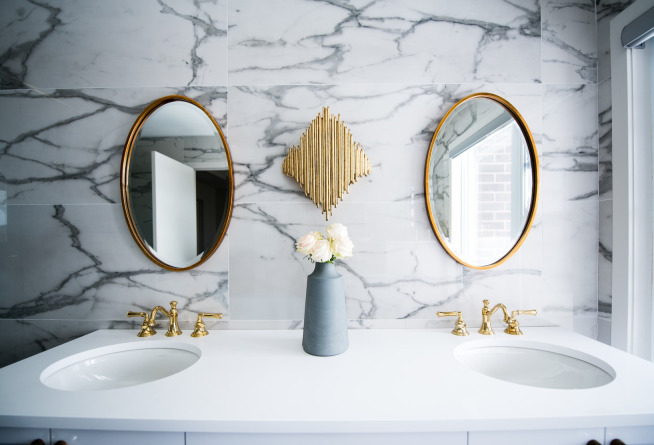
Photo: From Christian Mackie, Unsplash.

Apart from good preparation and serious, competent and proactive management of the crisis, another lesson at the country level came as a rough awakening to many nations when all borders between were closed during lockdowns. Suddenly, some countries realised how potentially dangerous it is to economically depend on other countries or on their citizens. Namely, when all countries closed their borders to travel at about the same time, it suddenly became very important to be self-sufficient. The basic needs for the preservation of human lives, health systems functioning, and keeping the economy going had all needed to be met from within [14].

One important lesson is that countries need to depend on their own food supply and production, rather than indulge mainly in cheaper food imported from other countries. Also, it is not reasonable to depend too much on the arrival of foreign tourists in the country. It would be wiser to develop other industries in parallel, that could also be competitive on a global scale. This pandemic showed that it is especially useful to develop digital industries. They can continue to operate during pandemics, their employees can work from their homes, and their products and services can still be sold around the world [15].

So, there were multiple underlying causes for the harm inflicted by this pandemic, some of them operating at the global level and some at the level of countries. But there are also lessons on a personal level. We are finding ourselves in an unexpected and still uncertain situation for months now. The first wave of the COVID-19 pandemic has already significantly changed everyone’s lives. The uncertainty is still ongoing, and most experts feared further waves. Many find it increasingly difficult to cope with this uncertainty, so no one should be judged too harshly in this difficult situation. There are still no clear solutions until we develop either medicines or vaccines that will be effective and safe enough.

However, they can only be developed by a tiny fraction of the entire world population – the scientists. Therefore, going back to the life as we knew it, where we shook hands, hugged and kissed, and lived between two big concerts, or sporting events, or restaurant dinners, or vacations – it all now depends on scientists. For much of the year 2020, we could only hope that there was a group of them working tirelessly to develop an effective vaccine or a useful drug somewhere in the world. Until then, we were only able to continue to live in a way that felt suboptimal to many, swaying between two bad choices.

However, it saddens what I predict will likely happen after the scientists succeed. When they manage to protect the rest of us from a new coronavirus – and I do hope that it will happen over time – I fear the speed at which people will continue to live as if nothing had even happened. This pandemic may soon fall into oblivion. But all its causes, listed in this article, will remain present. This will ensure that there is fertile ground for the onset of a new pandemic in this century, which could have much more severe consequences. It could have a higher death rate, spread even more efficiently and affect younger age groups.

The pandemic showed us the need for close collaboration between science and governments, but also for the responsible role of the media. The advice that governments and the general public were getting from scientists was invaluable in shaping the national response in many countries. The more the governments adhered to evidence-based methods and policies, the more successful they were in their protection of human lives, their economic activities and national security. It seems reasonable, following this pandemic, to demand greater investments into the diversity of microbes globally and their pathogenic potential. There is continuing need to explore global threats and provide better evidence for decision-making should we encounter an even more dangerous pandemic in the future. Decisive experiments should be funded, that could deliver actionable evidence.

In the aftermath of this pandemic, are we going to learn all these lessons and significantly change our ways, as humanity? Will we eradicate hunger, work to preserve species biodiversity, start investing more in prevention, and work actively to alleviate inequality? Will states begin to think strategically and conserve resources within their borders that guarantee their self-sufficiency in the event of a new pandemic? Will people generally start judging each other less harshly, show more solidarity with those less fortunate, cooperate and support each other more?

Unfortunately, I do not see that outcome as likely. Rather, I would predict that most people will hardly wait to return to their previous way of life, leading to even greater inequalities and further biodiversity loss, with a persistent hunger problem among the world’s poorest. The value of investment in prevention will continue to be underestimated. Many countries will continue to develop their economies in a way that is easy for their leaders, instead of being wise and forward-thinking. Individuals will continue to perceive themselves as competitors to each other and judge each other harshly.

That is why I predict that in the coming decades we will continue to be exposed to the dangers of pandemics and that in time something even more dangerous and devastating than the seventh coronavirus could hit us. Thus, even when science protects us from the threats from COVID-19, this is unlikely to mark the end of our fight against pandemics in the 21st century. It will only be the end of the beginning.
